# Effects of the COVID-19 pandemic on reading performance of second grade children in Germany

**DOI:** 10.1007/s11145-022-10379-y

**Published:** 2022-11-16

**Authors:** Natalie Förster, Boris Forthmann, Mitja D. Back, Elmar Souvignier

**Affiliations:** Department of Psychology, University of Münster, Fliednerstraße 21, 48149 Münster, Germany

**Keywords:** COVID-19 pandemic, Inequality, Student performance, Learning progress assessment, Reading

## Abstract

In education, among the most anticipated consequences of the COVID-19 pandemic are that student performance will stagnate or decline and that existing inequities will increase. Although some studies suggest a decline in student performance and widening learning gaps, the picture is less clear than expected. In this study, we add to the existing literature on the effects of the COVID-19 pandemic on student achievement. Specifically, we provide an analysis of the short- and mid-term effects of the pandemic on second grade reading performance in Germany using longitudinal assessments from over 19,500 students with eight measurement points in each school year. Interestingly, the effects of the pandemic established over time. Students in the first pandemic cohort even outperformed students from the pre-pandemic cohorts and showed a tendency towards decreased variances during the first lockdown. The second pandemic cohort showed no systematic mean differences, but generally had larger interindividual differences as compared to the pre-pandemic cohorts. While the gender achievement gap seemed unaffected by the pandemic, the gap between students with and without a migration background widened over time—though even before the pandemic. These results underline the importance of considering effects of the pandemic across cohorts, large samples, and fine-grained assessments. We discuss our findings considering the context-specific educational challenges and in terms of practical implications for teachers’ professional development.

## Introduction

After 2 years of the COVID-19 pandemic, more than 6 million people have lost their lives according to official statistics (Johns Hopkins University, n.d.). This tragedy has been accompanied by massive restrictions on fundamental rights including the freedom of movement, the freedom of assembly, and the freedom of occupation. It has also affected the right to education. As governments across the world have taken necessary steps to limit the spread of the coronavirus and announced the shutdown of schools, more than 1.5 billion students (90%) in over 190 countries had to stay at home (UNESCO, 2020). The world has never experienced such a dramatic impact on global education, and concerns about long-term educational, economical, and social consequences are high.

In education, among the most anticipated consequences of the COVID-19 pandemic are that student achievement will stagnate or decline and that existing inequities will increase (Helm et al., 2021; Kuhfeld et al., 2020a; Leopoldina, 2020; Wößmann, 2020). Indeed, some studies have shown that these concerns may be justified, pointing to a decline in student performance and a widening of learning gaps because of pandemic-related school closures (Engzell et al., 2021; Hammerstein et al., 2021; Ludewig et al., 2022; Werner & Wößmann, 2021). However, other findings suggest that there may have been surprisingly little or no loss of learning, and that even low-performing students may have reached achievement levels similar to those of low-performing students prior to the pandemic (e.g., Depping et al., 2021; Gore et al., 2021; Kuhfeld et al., 2020b). Yet, most of these studies compare student achievement after school closures at only one specific measurement point (e.g., at fall 2020) to student achievement before school closures (e.g., at fall 2019). Insights into the dynamics during the pandemic are currently lacking. Similarly, there are as yet hardly any findings for very young students at the start of primary school. For Germany in particular, the current findings are based on national or international comparative studies with very large samples, but some of the comparative data from before the pandemic are several years old (Ludewig et al., 2022) or were collected at a different point in the school year (Depping et al., 2021).

To get a comprehensive picture about the (cumulative) effects of the COVID-19 pandemic in education, it is necessary to examine effects for different countries, subjects, and age groups over time because (a) educational systems and their teachers were differently prepared for distance teaching particularly at the beginning (Huber & Helm, 2020; OECD, 2020), (b) children are more likely to read than do math or write in their free time or when schools are closed (Gunzenhauser et al., 2021; Werner & Wößmann, 2021; Wößmann, 2020), and (c) distance learning requires a high degree of self-regulatory skills, which are less developed in younger children compared to older children (Blume et al., 2021; Roebers, 2017).

In this study, we add to the existing literature on the effects of the COVID-19 pandemic in education by providing a differentiated analysis of the short- and mid-term effects of the pandemic on second grade reading performance in Germany. We provide a more detailed examination of student learning within the two school years affected by the pandemic, 2019/20 and 2020/21, compared to pre-pandemic cohorts 2015/16 till 2018/19 by analyzing highly sensitive longitudinal assessments with eight measurement points in each school year on a large sample of 19,654 second-grade students.

### Teaching in Germany during the COVID-19 pandemic

As in most other countries, one of the first immediate policy responses to the COVID-19 pandemic in Germany was to close schools to prevent infections and contain the spread of the coronavirus. After the first pandemic wave with school closures of about 11 weeks on average in spring 2020 for students from first to third grade, the new school year in summer 2020 began with in-person learning. However, due to subsequent pandemic waves, schools were closed again in December 2020 and early 2021. Thus, starting in March 2020, instruction varied between distance-only instruction (in periods of school closures), face-to-face instruction with spacing rules and masks (in periods of relatively low incidence), and alternating instruction, in which half of the class attended school for a week and then received a week of distance instruction. In addition to these general politically-regulated school openings and closings, individual schools needed to be closed following COVID-19 outbreaks. Likewise, individual students sometimes had to stay at home due to quarantine regulations.

These protective measures adopted for schools because of the pandemic and associated concerns about the short- and long-term consequences for students led to an increase in educational research on this topic. First, surveys of teachers, parents, and students were available, highlighting the enormous educational challenges posed by the pandemic (e.g., Fickermann & Edelstein, 2020; Helm et al., 2021; Wößmann, 2020). For Germany, the findings of these early studies reflect what was stressed by an OECD report (2020) at the very beginning: A major problem during the school shutdown was that German teachers and the German educational system were poorly prepared to break new digital ground in teaching. According to OECD statistics, German teachers’ technical and pedagogical skills for integrating digital devices in instruction, their time for preparing lessons that integrate digital devices, and their effective professional resources for learning how to use digital devices were far below OECD average (OECD, 2020). Moreover, more than 50% of the German schools lacked sufficient qualified technical assistant staff and an effective online learning support platform. Unsurprisingly, more than half of the teachers reported that they were not or were badly prepared for distance teaching and 10–30% judged their own and their colleagues’ competencies for distance teaching to be insufficient (Helm et al., 2021).

Against this background, reports from parents indicate that students received very little formal school-based instruction during times of school closures, particularly at the beginning of the pandemic and particularly at elementary schools (Wößmann et al., 2020, 2021). According to findings from Wößmann et al. (2020), more than half of the students in Germany (57%) had online lessons less than once a week and only 6% had online classes daily. While learning activities decreased severely during the period of school closures, activities such as watching TV, playing computer games and using cell phones increased (Grewenig et al., 2020; Werner & Wößmann, 2021; Wößmann et al., 2020). Unsurprisingly, parents expressed concern that their children were not learning enough during the time of the school closures (Helm et al., 2021). However, during lockdowns, many schools focused on the subjects German and mathematics at the expense of other subjects such as science, art, music, and sports (Wildemann & Hosenfeld, 2020). In addition, students spent more time on leisure activities that are more conducive to student development such as reading, creative work, and exercises (Wößmann et al., 2020). Thus, whether school closures impaired students’ development depends not only on the subject and the amount of formal school-based instruction but also on which alternative activities were carried out.

Another important aspect is the amount of parental support students received, as this might compensate for missing teacher-based instruction (Gunzenhauser et al., 2021). As Martin-Chang et al. (2011) showed, the academic achievement of students who learn at home depends on whether structured or unstructured homeschooling (also referred to as unschooling or autonomous learning) occurs. While students receiving structured homeschooling showed higher academic achievement than students learning at school, academic performance was lower in the unstructured homeschooling group compared to both other groups. In Germany, school attendance is compulsory, and most parents with minor children are employed part time or full time (Statista, 2022), and are, presumably, ill-prepared to teach. Thus, the need for distance learning was unintentional and learning at home most likely resembled the unschooling or autonomous learning condition (Helm et al., 2021). Although parental support while learning increased by half an hour during the shutdown, students needed to self-regulate their learning most of the time (Wößmann et al., 2020), which poses a great challenge particularly for younger students (Blume et al., 2021; Roebers, 2017). Therefore, it seems unlikely that the unfavorable conditions of distance learning could be fully compensated by parental support.

A closer look at these first survey data from teachers and parents gives reasons for further concerns. For example, Wößmann et al. (2020) found that students differed in the amount of time they spent on school-related activities, as well as on conducive and detrimental leisure activities, depending on their gender and their prior skills to the disadvantage of boys and lower-performing children. While social inequalities generally explain much of the variance in students’ academic achievement (Hußmann et al., 2017; Mullis et al., 2017), differences in school-related activities during school closures were surprisingly less pronounced between students with highly educated and less-educated parents (Grewenig et al., 2020; Wößmann et al., 2020). Still, there is reason to be concerned that performance gaps between boys and girls and between students who were already struggling before the pandemic and students who were performing well may have widened further due to the unfavorable learning conditions.

### Reading performance and reading instruction in Germany before the pandemic

To compare cohorts that were affected by COVID-19-related school closures and those that were not, we need to consider what reading achievement and school-based reading instruction looked like in Germany before the pandemic. According to the latest results of the *Progress in International Reading Literacy Study* (PIRLS), German fourth graders achieved about average reading skills compared to the average achievement in the EU or other OECD countries (Mullis et al., 2017). Yet, variance between students reading skills was strikingly high and exceeds the between-students variability of a great majority of participating European countries (Bos et al., 2017). Most importantly, however, student reading performance in Germany has always been strongly related to socio-economic status (SES), and this influence has even increased significantly in reading since 2001, leading to Germany being among the states with the strongest relation between SES and student reading performance. Similarly, comparatively large differences in reading skills between students with and without a migration background were regularly found in Germany (Wendt & Schwippert, 2017). This finding is not surprising, as a migration background is often confounded with a lower socio-economic status (Hippmann et al., 2019; Stanat et al., 2010). However, in times of school closures when students need more parental support, the fact that immigrant parents do not speak the language of instruction may have a greater impact than socioeconomic status in widening this gap.

The PIRLS 2016 data for Germany also showed that girls systematically outperform boys. One explanation for this superiority could lie in reading motivation and reading behavior, which are significantly related to reading achievement (Becker & McElvany, 2018; Hebbecker et al., 2019). As girls are more motivated to read, they are more likely to read in their leisure time compared to boys (e.g., Becker & McElvany, 2018; Lepper et al., 2021; McElvany et al., 2017). Contrary to the achievement gap for students with different SES, the achievement gap favoring girls is about the same in Germany as in other EU and OECD countries overall (McElvany et al., 2017). Thus, the main problem with students’ reading performance in Germany is not underachievement but rather large interindividual differences in achievement and strong correlations with social status and migration background, indicating that strong social inequalities in education already existed in Germany before the COVID-19 pandemic. Moreover, evidence suggests that the conditions were more favorable for girls and for students without a migration background to cope with the pandemic in terms of reading development.

Regarding reading instruction, PIRLS data showed that in Germany, both the absolute and relative time explicitly devoted to reading instruction is well below the international average (Bremerich-Vos et al., 2017). Lower rates were found in only six participating countries and regions. More detailed insights into the business-as-usual reading instruction for German second graders are provided in a recent study by Peters et al. (2022), who observed the reading instruction of 52 classes. They found that the typical reading instruction can be characterized as being mainly teacher centered and that students also worked individually and silently with mostly continuous texts. Apart from giving feedback, evidence-based elements of effective reading instruction were rarely observed. Thus, findings from both large-scale assessments and observational studies suggest that reading instruction in Germany before the pandemic was suboptimal in terms of both the amount of time spent and the methods used.

In sum, the educational system and German teachers were poorly prepared for distance teaching when the pandemic commenced. As a result, instructional time was short especially during school closures, and was hardly compensated by parents, raising concerns that student learning was impaired. These changes in learning-related and leisure time activities, however, differed between boys and girls and higher- and lower-performing students. In addition, parents who do not speak the language of instruction may have had particular difficulty compensating for school closures. Thus, the pandemic might have served as an engine of social inequality by increasing educational inequality. Yet, increased levels of leisure activities such as reading and the fact that reading instruction in German schools was also not ideal before the pandemic, may have mitigated these negative effects, particularly for girls and high-achieving students.

### Previous findings on the effects of COVID-19-related school closures on student performance

Shortly after a wave of descriptive studies about student learning during the pandemic, researchers very quickly published initial studies on the effects of pandemic-related school closures, which have been summarized in reviews and meta-analyses (Donnelly & Patrinos, 2021; Hammerstein et al., 2021; Panagouli et al., 2021; Zierer, 2021). One of the first overviews was provided by Hammerstein et al. (2021) who reviewed *n* = 11 studies from seven different countries which mostly investigated reading and mathematics. They concluded that the pandemic led to overall negative effects on student achievement (*Mdn* = − 0.08 *SD*) and that these negative effects were more pronounced for younger students (see also Georgiou, 2021) and students with low SES. However, effects ranged from − 0.37 *SD* to + 0.25 *SD* and two of the studies with positive effects on student achievement included reading (Depping et al., 2021; Gore et al., 2021). Other findings also indicate that learning losses varied by subject and were typically smaller for reading than for mathematics (e.g., Zierer, 2021). Recently, König and Frey (2022) performed a meta-analysis including 18 studies with a total of 109 effect sizes. Their results are well in line with the findings of Hammerstein et al. (2021), in that they showed a robust negative average effect of *d* = − 0.18 (*SE* = 0.063). Again, younger students appeared to be more negatively affected by lockdowns. Interestingly, results indicate that the negative effect was reduced upon later school closures in winter 2020 and in the beginning of 2021 (see also Schult et al., 2022).

To date, four studies have reported findings on the effects of COVID-19 related school-closures on student reading achievement in Germany (Depping et al., 2021; Ludewig et al., 2022; Schult & Wagner, 2021; Schult et al., 2022). Depping et al. (2021) compared data from Hamburg elementary schools assessed in April and May of 2019 (end of third grade) with data from August and September of 2020 (beginning of fourth grade) as well as from secondary schools (beginning of fifth grade 2019 vs. 2020) using the same reading tasks. In both comparisons, they found no significant differences between the two cohorts. Students from secondary schools even showed a tendency toward higher reading comprehension. However, more students in the pandemic cohorts had to be excluded from the analyses because they completed fewer tasks than needed to reliably estimate proficiency, and this exclusion was higher when more students came from lower socio-economic backgrounds.

In two studies, Schult and colleagues presented short- and long-term effects of the temporary school closures in Germany for secondary schools in Baden-Württemberg. In their first study (Schult & Wagner, 2021), they reported that reading competencies were slightly lower (− 0.07 SD) in 2020 compared with the competence levels from the three previous years, but low-performing students achieved similar competence levels as in previous cohorts. Moreover, socio-cultural capital and the proportion of students with a migration background played only a minor role in explaining learning loss. By contrast, in 2021, reading proficiency was quite similar to that of the pre-pandemic cohorts, as indicated by a standardized mean difference of − 0.02. And, in the long run, school closures negatively affected low-achieving students learning, and schools that were already disadvantaged in terms of the social capital of their students suffered greater learning losses. This picture was less pronounced in reading than in mathematics (Schult et al., 2022).

Only recently, Ludewig et al. (2022) presented their findings on COVID-19 effects using representative PIRLS data. As expected, they found that students’ reading achievement was lower in 2021 than in 2016, with a difference equivalent to about one-third of a year’s learning growth. In contrast to their hypotheses, however, none of the achievement gaps (namely, between students with different socio-cultural capital, with and without a migration background, and between boys and girls) had widened significantly between 2016 and 2021.

All four German studies investigated students’ reading skills at the end of primary school or at the beginning of secondary school and are based on large datasets that are representative for the state of Hamburg or Baden-Württemberg or even for Germany. Similar to the designs of international studies, in each of the German studies, student performance after the pandemic is compared to the performance of one similar cohort from before the pandemic at a specific point in time, mostly at the beginning of the school year after the summer holidays. In Depping et al.’s study (2021), some of the data in the pandemic cohort could not be collected in the same month and therefore not in the same school year, so the performance of third graders from April/May 2019 was compared to the performance of fourth graders from August/September 2020. Ludewig et al. (2022) compared PIRLS data from 2021 to PIRLS data from 2016, thus using a five year old cohort for comparisons.

In sum, one of the most anticipated consequences of the COVID-19 pandemic in education is that student learning has been negatively affected by school closures and that existing inequities increased. While survey data on distance learning and teacher support fuel these concerns, empirical data on student achievement to date suggest that, at least in reading, the impact may be not as negative as expected. The results available to date come from studies that primarily examined students from end of primary school or from fifth grade onward at a single measurement point during the school year, often directly after the summer holidays.

### The present study

A significant portion of school shutdowns in Germany occurred during the 2020 Easter vacations and 2020 Christmas vacations, as well as in the weeks following. Decreasing student achievement and increasing variability of student performance, however, is discussed as an effect of vacation (e.g., Dumont & Ready, 2019). To reliably estimate the effects of the COVID-19 pandemic on student achievement and achievement inequality, it is critical to separate typical developmental effects and effects of vacation from unique effects of the shutdown. Multiple measurements throughout the school year would provide more accurate insights into the dynamics of the pandemic’s effects. In this study, we thus examine the average performance, interindividual differences in performance, and performance gaps between boys and girls and between students with and without a migration background at eight measurement points using learning progress assessments (LPA) during the COVID school years 2019/20 and 2020/21 and compare it with data from the four school years before. We investigate the reading skills of students in second grade using sample sizes of over 4000 students for each cohort to differentiate typical performance and performance variability in cohorts before the pandemic from unique effects likely caused by the pandemic. Because schools were repeatedly closed, but also because instruction could not proceed as usual when schools were open, we expected the following:

First, we assumed that student performance would be lower in the two pandemic cohorts after the shutdowns compared to the cohorts before the pandemic (Hypothesis 1).

Second, we expected that interindividual differences between students increased because of the pandemic (Hypothesis 2).

Finally, we hypothesized that performance gaps would be higher between boys and girls (Hypothesis 3a) and students with and without a migration background (Hypothesis 3b) in the two pandemic cohorts than in the years before the pandemic. These hypotheses were not preregistered.

## Method

### Participants

We used data from second graders from four pre-pandemic cohorts assessed between the school years 2015/16 to 2018/19 (*N* = 12,037 students; *n* = 636 classes; *n* = 250 schools) and from the two cohorts that were (partly) affected by the pandemic in 2019/20 (*N* = 6,379 students; *n* = 338 classes; *n* = 182 schools) and 2020/21 (*N* = 5,284 students; *n* = 262 classes; *n* = 142 schools). For the 2019/20 cohort, the first four assessments were completed before the pandemic, the fifth assessment was partly completed before and partly during the first weeks of the first lockdown and the last three assessments were conducted during or after the school closures in Germany. For clarity, we compare students reading performance and variance in reading performance of the first and second pandemic cohorts with those of all four pre-pandemic cohorts below. However, we also cross-checked all comparisons for each of the two pandemic cohorts with each of the four pre-pandemic cohorts separately. Results are similar to those reported below. The detailed analyses for all separate comparisons can be found in the Supplemental Material (SM) 5 (https://osf.io/vphyt/).

Before data analysis, we excluded students who were younger than 6 years (*N* = 22) or older than 12 years (*N* = 443), students who completed the tests for second grade but were in first or third grade (*N* = 723), students with missing values at all measurement occasions (*N* = 1988), and students who were identified as duplicate cases (e.g., those who repeated second grade and, thus, participated twice in the assessment; *N* = 870). The total sample used in this study consisted of *N* = 19,654 second grade students, of which *N* = 5221 were from the first pandemic cohort and *N* = 4172 were from the second pandemic cohort. Detailed sample information can be found in Table 1. The decision to use the learning progress assessment in the classroom was made voluntarily by the teachers. As the learning progress assessment is used primarily in Hesse and North Rhine-Westphalia, most data (88.62%) came from these states. The use of the completely anonymized data for scientific purposes is contractually regulated, has been certified by a data protection officer, and complies with the ethical standards of the German Psychological Society and the Association of German Psychologists.

**Table 1:** Descriptive characteristics of the samples

	Age			Gender		Migration background	
	*M*	*SD*	% NA^a^	% Female	% NA	% Yes	% NA
Pre-pandemic cohorts (*N* = 10,261)	7.93	0.49	35.35	47.94	0.00	24.32	0.16
First pandemic cohort (*N* = 5221)	7.96	0.49	46.33	46.22	0.00	23.96	0.00
Second pandemic cohort (*N* = 4172)	7.93	0.59	53.04	46.24	0.00	24.11	0.00
Overall (*N* = 19,654)	7.94	0.51	42.02	47.12	0.00	24.18	0.08

^a^NA = missing values

### Measures

#### Learning progress assessment

Data were collected with the web-based platform quop (Souvignier et al., 2021) using the second grade reading test series quop-L2. This test series consists of four equivalent tests that measure component processes of reading comprehension at the word, sentence, and text levels (Förster & Kuhn, 2021; Förster et al., 2021). At the word level, students must decide if the word shown is a real word in the German language. At the sentence level, students decide whether a sentence (e.g., Ice is hot.) makes sense, and at the text level students must indicate if a third sentence completes two prior sentences in a meaningful way. In total, each of the four tests consists of 20 word items, 13 sentence items, and 13 text items. Items were developed using strict design rules, resulting in four equivalent test forms for the test series (Förster & Kuhn, 2021). All answer formats are *yes* or *no*, and for each answer the system records both accuracy and response time. Items are provided without time limit, but students are instructed to answer all items as accurately and quickly as possible. On average, students needed 9.17 min (median time) to complete the tests at the beginning of the school year and 4.28 min (median time) at the end of the school year. The four tests were further divided into test halves and recombined to construct another four parallel tests based on the same item pool. To prevent confounding of item and time of measurement, classes in each school year were randomly assigned to one of eight groups, where each group completed a different combination of test halves per time point. Over the first four time points, each group completed each item once. To assess the reading progress across the school year, the same four tests were repeated for timepoints five to eight (see SM 1 for a description and visualization of the study design). The test series has been shown to be reliable and valid for assessing student reading progress (Förster et al., 2021). Measurement invariance was found across time (Förster et al., 2021; Forthmann et al., 2022) and across cohorts, migration background, and gender (see below). Immediately after test completion, teachers could see the performance of the individual students and the whole class via a teacher platform. Prior to the pandemic, students completed the computer-based tests exclusively at school during self-study periods or as a group test in a computer pool. When schools were closed, teachers were explicitly instructed to have students take the tests at home to monitor their progress during remote learning. We provided specific instructional materials explaining how to administer the tests at home, how parents should behave during the tests, and why it was important that they not help their children as this would affect the measurement and interfere with teachers’ decision-making based on the data.

#### Demographic variables

Students’ gender and migration background were entered by teachers in the quop system. Gender was coded male = 0 and female = 1; no other gender than male or female was present in the data. Students without a migration background were coded 0, and students with a migration background were coded 1. Migration background can be defined differently, e.g., by students’ or parents’ country of birth, students’ mother tongue, or students’ family language. The quop system provides no explicit definition of migration background. Thus, teachers will most likely record a migration background for those students whose migration background is noted in official school records as well as for those whose migration background they deem relevant for data interpretation.

### Data analysis

Data preparation and all analyses were performed with the open-source software R (version 4.0.2 R Core Team, 2020). We have provided the scripts and all data necessary to replicate the analyses in the OSF (https://osf.io/vphyt/).

#### Scoring of the learning progress assessment

Students’ reading performance was modeled as the efficiency of reading comprehension processes. To this aim, we combined accuracy and response time information of the quop-L2 test series by computing the correct item summed residual time (CISRT) proposed by van der Maas and Wagenmakers (2005; for a detailed argumentation for the CISRT score see Forthmann et al., 2022). In the CISRT scoring procedure, incorrect responses are awarded zero points. For correct responses, the time difference between the response and a time limit is calculated, the greater the time difference between the response and the time limit, the higher the score for that response. Thus, correct answers are rewarded more the faster the answer was given. Given that the quop tests themselves do not include a time limit, we set a time limit based on the response time distribution. For each scale, we defined cutoffs at the 5% and 99.5% latency quantiles. Any response times faster than the 5% quantile were recorded to be missing to correct extreme response times due to fast guessing. The 99.5% latency quantile was used as the time limit to calculate the CISRT scores. Any response times above this cutoff were also recorded to be missing (see SM 1 for details on the exact calculation procedure, including the definition of time limits).

#### Models

In the absence of specific hypotheses about the effects of the pandemic on reading skills at the word, sentence, and text levels, we modeled the overall reading performance at each point of measurement as a latent variable based on the three scales as observed indicators (c.f., Forthmann et al., 2022).^1^ The full measurement model can be found in SM 2. The loading for the sentence score was fixed to 1 at each point of measurement for model identification purposes, while the loadings for the word and text scores were freely estimated. This resulted in large, standardized loadings ranging from 0.72 to 0.75 for word level, 0.79 to 0.81 for sentence level, and 0.61 to 0.65 for text level (cf. Kline, 2005).

To allow for comparisons between the different cohorts, we first checked whether measurements were strictly invariant across cohorts (for detailed results, please see SM 3). Based on the result of strict measurement invariance across cohorts, we then constrained the loadings for the word and text scores to be equal across time to build a longitudinally weak invariance model, which is sufficient for interpreting latent variance across time (Hypothesis 2). To interpret latent means across time (Hypothesis 1), a model with longitudinally strong measurement invariance is needed. We thus constrained the intercepts of all three indicators to be equal across time. All error variances of observed variables were estimated freely. Residual covariances of the same level-of-language score were estimated for the word and text levels, but not for the sentence level (for comparisons between models with differently modeled residual covariances, see SM 3 in the OSF). Latent factor means, latent factor variances, and all inter-factor covariances were estimated freely. While for Hypothesis 1 strong measurement invariance across the three groups would have been sufficient and Hypothesis 2 could have been tested based on a between-group weak measurement invariance model, the much stronger levels of longitudinal invariance of the models used for hypothesis testing further allow us to interpret the learning progress and the variance changes across time in each of the groups.

Testing of Hypotheses 3a and 3b required further evaluation of measurement invariance. Specifically, for Hypothesis 3a, we needed to establish strong measurement invariance between all groups resulting from the group × gender interaction (i.e., splitting for boys vs. girls in the pre-pandemic cohorts and each of the pandemic cohorts). In addition, testing Hypothesis 3b required strong measurement invariance between all groups resulting from the group × migration background interaction (i.e., splitting for migration background-no vs. -yes in the pre-pandemic cohorts and each of the pandemic cohorts). Detailed measurement invariance results for these additional checks are available in SM 3 in the OSF.

In sum, the test of Hypothesis 1 was based on a longitudinally strong invariance model (χ^2^(699) = 2294.95, *p* < .001; RMSEA = .025, 90%-CI: [.024, .026]; SRMR = .052; CFI = .984; TLI = .981), Hypothesis 2 was tested based on a longitudinally weak invariance model (χ^2^(630) = 1746.13, *p* < .001; RMSEA = .022, 90%-CI: [.021, .023]; SRMR = .048; CFI = .989; TLI = .985), and Hypotheses 3a and 3b were tested based on a model with strong invariance between the respective groups (gender gap: χ^2^(1160) = 1855.38, *p* < .001; RMSEA = .017, 90%-CI: [.015, .018]; SRMR = .034; CFI = .994; TLI = .992; migration background gap-ranges across separately fit models: RMSEAs ranged from .022 to .023; SRMRs ranged from .043 to .059; CFIs ranged from .988 to .990; TLIs ranged from .985 to .987). For Hypothesis 1, the latent mean differences between cohorts were estimated in the effect size (ES) metric of Cohen’s *d*_*av*_ (e.g., Lakens, 2013), i.e., the latent mean difference was divided by the average of the respective latent standard deviations. For Hypothesis 2, we report the raw variance differences between the respective cohorts. Finally, for Hypothesis 3a and 3b, we first report Cohen’s *d*_*av*_ between gender or migration background groups in each of the three cohorts and at each measurement point. Next, the differences in *d*_*av*_ values between the pre-pandemic cohorts and both pandemic cohorts, respectively, were examined. All these differences (i.e., in latent means, latent variances, and latent differences) were estimated as newly defined parameters in the R package lavaan (Rosseel, 2012). Standard errors for such newly defined parameters in lavaan are based on the delta-method (Dorfman, 1938). As in previous work in which quop-L2 was scored by CISRT (e.g., Förster et al., 2021; Forthmann et al., 2022), all CFA models were estimated by means of robust maximum likelihood to account for multivariate non-normality. In addition, we took the nested data structure (students nested in classes) into account by requesting robust standard errors via the cluster argument in lavaan’s cfa() function.

#### Missing values

Overall, 32.66% of the data were missing. More data were missing in the two pandemic cohorts (pre-pandemic: 17.29%; first pandemic cohort: 49.77%; second pandemic cohort: 48.80%) and at later measurement points in each school year. We checked the missing data pattern and found that a MCAR pattern could not be reasonably assumed based on Jamshidian and Jalal’s two-step procedure (Jamshidian et al., 2014). However, when creating 28 equally sized bins for a simple total score at T1 (i.e., T1 reading performance is statistically controlled to a certain degree), we found that in 78.6% of the bins the MCAR assumption could not be refuted (for a similar approach, see Hebbecker et al., 2022). We thus assume that the data follow a MAR pattern, and that the missing data pattern is most likely more on the MAR side of the continuum of missing data patterns between MAR and MNAR (Graham, 2009; Newman, 2014). Consequently, we used full information maximum likelihood estimation to deal with missingness (c.f., Asendorpf et al., 2014).

While the amount of missing data was similar in both pandemic cohorts, the missing data in the first pandemic cohort occurred primarily at the time of the school closures. In contrast, in the second pandemic cohort, missing data were more evenly distributed across the school year. For the first pandemic cohort, the massive increase in missing data for the last points of measurement, resulted in the covariance coverage being very low, as noted during model estimation. Hence, to ensure that this identified issue does not affect our results and conclusions, a robustness check was carried out for the first pandemic cohort, which we report in detail for each of the evaluated hypotheses in SM 4 in the OSF. Specifically, we reduced participants in the first pandemic cohort to subsets that fulfilled minimal criterial with respect to missing values. We then applied a matching procedure to include further participants from the pre-pandemic cohorts that optimally matched participants in the respective subsets according to reading performance at the first measurement point, number of available measurement points, and gender. Importantly, results for Hypothesis 1 and Hypothesis 2 did not differ for this alternative approach, which successfully prevented the issue with covariance coverage. For Hypotheses 3a and 3b, a similar matching approach was carried out within each of the respective groups (e.g., the group of female participants or the group of children with migration background). For a migration background, this was important to check because otherwise the only way to obtain technically sound findings (i.e., estimated models without flagging technical issues) was to fix latent variable covariances to be equal across groups (reported here in the paper). Technical problems arose from the comparably low relative frequency of children with a migration background in each of the respective groups (see Table 1). All details on the matching approach, relevant R code, and alternative results can be found in SM 4 in the OSF.

## Results

**Fig. 1:**
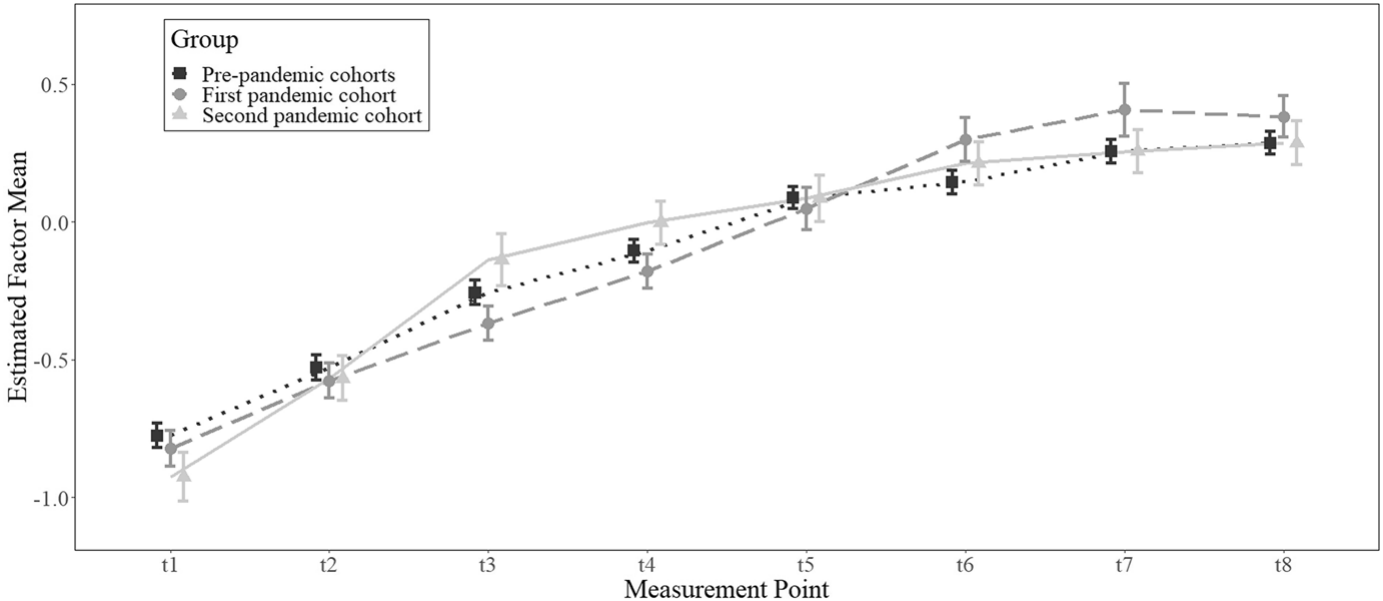
Estimated factor means over time. *Note* Error bars are 95% confidence intervals. Points at each measurement point are slightly shifted to facilitate interpretation

Figure 1 shows the learning progress (i.e., the latent means) over time for the pre-pandemic cohorts compared to the first and second pandemic cohort. Reading comprehension increased over time up to the seventh measurement point in all three groups. In the first pandemic cohort, there was a slight decrease towards the eighth measurement point. Yet, the overall learning progress trajectories indicate a learning plateau at the seventh measurement point.

**Fig. 2:**
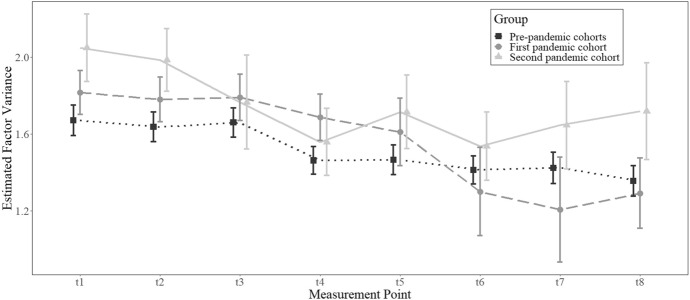
Estimated factor variances over time. *Note* Error bars are 95% confidence intervals. Points at each measurement point are slightly shifted to facilitate interpretation

Interindividual variability over time in the pre-pandemic and pandemic cohorts is shown in Fig. 2. Overall, the latent variances indicate that interindividual differences reduced over time, particularly in the pre-pandemic cohorts and the first pandemic cohort. For the second pandemic cohort, interindividual differences tended to be higher at most measurement points and a more unsystematic pattern with increasing variances for the later measurement points was observed.

### Performance differences between cohorts

**Fig. 3:**
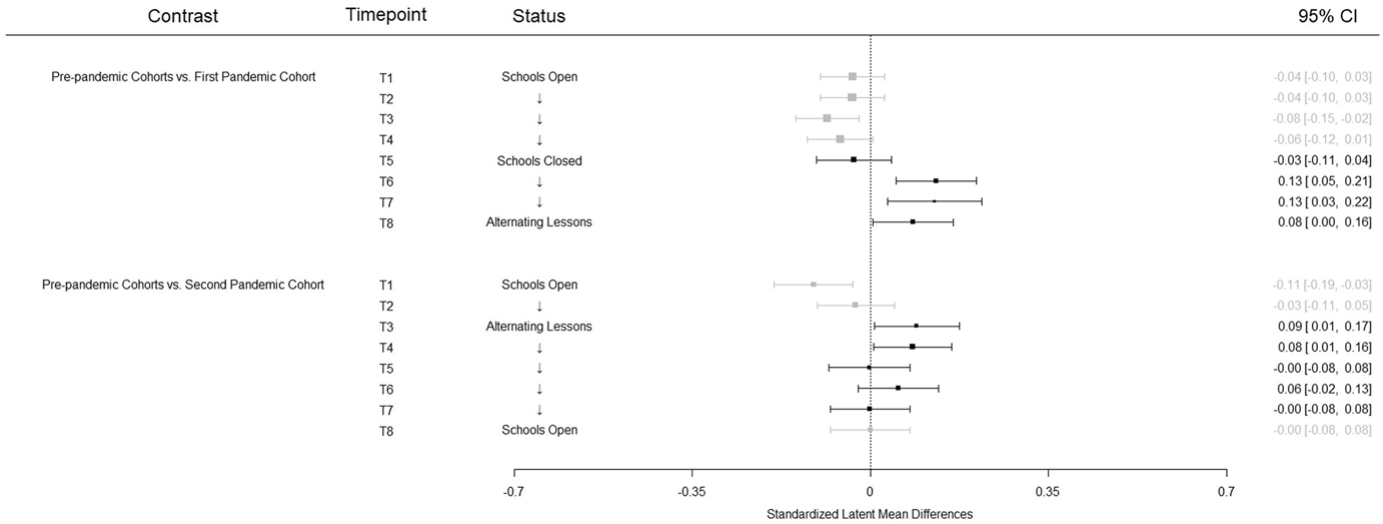
Standardized latent mean differences based on a longitudinally strong measurement invariance model. *Note* Measurement points affected by school closure or alternating lessons are depicted in black (and gray otherwise)

Regarding students' performance, we found unexpected results for the first pandemic cohort. While students in the school year 2019/20 started slightly lower compared to the pre-pandemic cohorts, they significantly outperformed the pre-pandemic cohorts during the first lockdown (see Fig. 3). These effects (based on latent means; *d*_*av*_) vary between ES 0.08 and 0.13. Also contrary to our hypothesis, the second pandemic cohort showed no systematically lower performance compared to the pre-pandemic cohorts. While students in the second pandemic cohort performed significantly worse at the beginning of the school year (ES = − 0.11), their performance exceeded that of the pre-pandemic cohorts around December 2020 and January 2021 (ES = 0.08 and 0.09) and showed no systematic differences (ES vary between 0.00 and 0.06) in the second half of the school year, when students returned to school (sometimes alternately). Overall, Hypothesis 1 was clearly not supported by the data.

### Interindividual differences in student performance

**Fig. 4:**
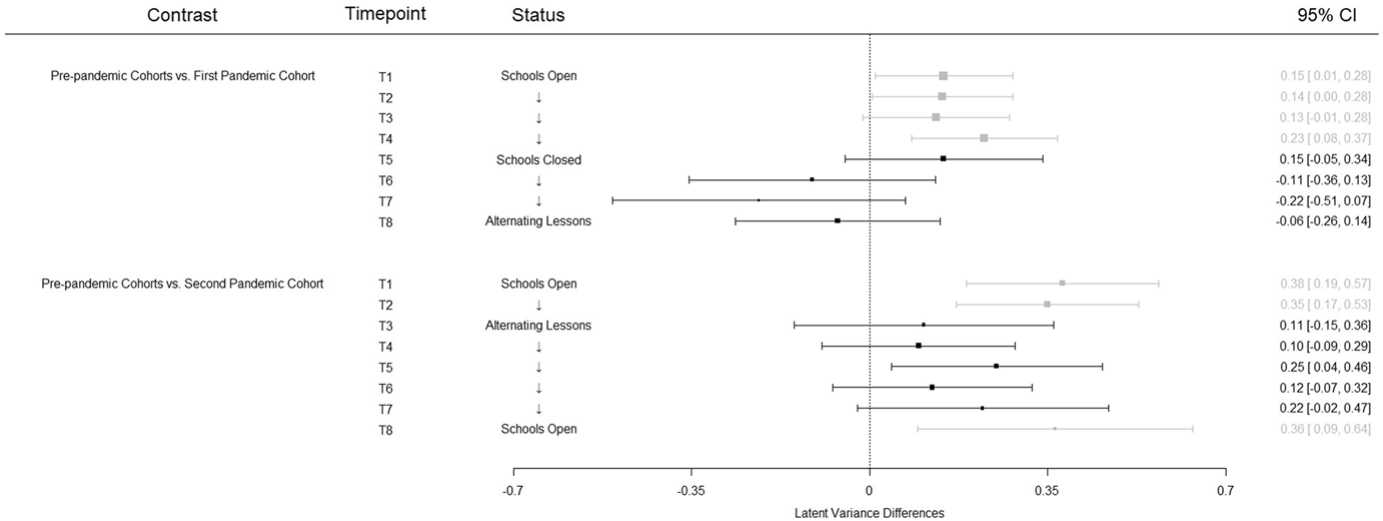
Latent variance differences based on a strict measurement invariance model across cohorts. *Note* Measurement points affected by school closure or alternating lessons are depicted in black (and gray otherwise)

The interindividual differences in the first pandemic cohort were consistently higher than in the pre-pandemic cohorts *before* the school closures and at three measurement points this difference was significant. During the first lockdown, however, the variances constantly decreased (see Fig. 4). Thus, this result pattern is the exact opposite of our hypothesis. In contrast, at the beginning of the second school year of the pandemic in 2020/21, students’ interindividual differences were significantly larger compared to the pre-pandemic cohorts. Although these differences weakened somewhat as the school year progressed, the overall pattern of these results is consistent with our Hypothesis 2, which stated that interindividual differences would be expected to increase because of the pandemic.

### Performance gaps

As expected, we found that girls outperformed boys in reading in all cohorts (see Table 2). In the pre-pandemic cohorts, the effect was significantly different from zero at all measurement points, and the effect size ranged from 0.09 to 0.14 (see Table 2). In the first and second pandemic cohorts, the range of gender gap effect sizes was quite similar, although the difference was non-significant at some of the measurement points. The highest gender gap effect size was observed for the second pandemic cohort (*d*_*av*_ = 0.18 at the third measurement point). None of the gender gap differences between the pre-pandemic cohorts and the first or second pandemic cohort were significantly different from zero. Thus, while the gender gap was replicated in this work, we did not find any evidence in support of Hypothesis 3a.

**Table 2:** Gender gap results

	Pre-pandemic cohorts		First pandemic cohort		Second pandemic cohort	
	ES	95%-CI	ES	95%-CI	ES	95%-CI
T1	0.09	[0.03, 0.15]	0.04	[− 0.05, 0.13]	0.11	[− 0.01, 0.22]
T2	0.09	[0.03, 0.16]	0.09	[0.00, 0.17]	0.09	[− 0.02, 0.19]
T3	0.09	[0.03, 0.15]	0.13	[0.05, 0.22]	**0.18**	**[0.05, 0.31]**
T4	0.12	[0.06, 0.18]	0.09	[0.00, 0.18]	**0.07**	**[**− **0.05, 0.18]**
T5	0.09	[0.03, 0.16]	**0.09**	**[**− **0.02, 0.20]**	**0.14**	**[0.02, 0.26]**
T6	0.14	[0.07, 0.20]	**0.13**	**[**− **0.03, 0.29]**	**0.09**	**[**− **0.03, 0.21]**
T7	0.11	[0.05, 0.18]	**0.04**	**[**− **0.12, 0.21]**	**0.13**	**[0.01, 0.25]**
T8	0.13	[0.07, 0.20]	**0.07**	**[**− **0.04, 0.19]**	0.10	[− 0.02, 0.22]
Average	0.11	[0.05, 0.16]	0.09	[0.00, 0.17]	0.11	[0.02, 0.21]

ES = Effect size (based on latent means, *d*_*av*_). Measurement points affected by school closure or alternating lessons are depicted in bold

Finally, we evaluated differences between cohorts with respect to the migration background gap. The migration background gap was generally supported by the data: In all cohorts and at all measurement points, students without a migration background significantly outperformed students with a migration background. The effect sizes ranged from 0.23 to 0.81 (see Table 3). The effect sizes in the first pandemic cohort were noticeably higher from the fifth measurement point onward (i.e., the first assessment affected by pandemic measures). Yet, when compared to the combined data of the pre-pandemic cohorts, the performance gaps in the first pandemic cohort were not significantly different at any point of measurement. In the second year of the pandemic, the performance gap varied between ES = 0.52 and 0.75. Compared to the performance gap in the pre-pandemic cohorts, it was significantly larger at three measurement points affected by pandemic measures (i.e., measurement points three, four, and six). However, the separate analysis of the four pre-pandemic cohorts revealed an important trend: Since 2015/16, the achievement gap between immigrant and non-immigrant students seems to have widened each year with a slight decrease from 2017/18 to 2018/19 (see Table 3). Hence, while the expected pattern of results, i.e., a widening of the achievement gap between immigrant and non-immigrant students was observed for the first pandemic cohort, the average gap slightly reduced in the second pandemic cohort. Moreover, as a tendency towards a widening of the achievement gap was observed in the four pre-pandemic cohorts, the separate impact of the pandemic on the increase of the achievement gap between students with and without a migration background remains unclear due to a confounding of this trend and the pandemic.

**Table 3:** Migration background gap results

	2015–16 Cohort		2016–17 Cohort		2017–18 Cohort		2018–19 Cohort		First pandemic cohort^a^		Second pandemic cohort	
	ES	95%-CI	ES	95%-CI	ES	95%-CI	ES	95%-CI	ES	95%-CI	ES	95%-CI
T1	0.23	[0.09, 0.37]	0.44	[0.08, 0.79]	0.53	[0.40, 0.67]	0.45	[0.34, 0.56]	0.56	[0.46, 0.67]	0.52	[0.38, 0.66]
T2	0.29	[0.15, 0.43]	0.34	[0.01, 0.68]	0.54	[0.41, 0.68]	0.49	[0.38, 0.60]	0.61	[0.51, 0.72]	0.54	[0.40, 0.69]
T3	0.24	[0.09, 0.39]	0.54	[0.19, 0.90]	0.53	[0.40, 0.66]	0.49	[0.38, 0.60]	0.59	[0.49, 0.70]	**0.75**	**[0.56, 0.94]**^b^
T4	0.24	[0.10, 0.39]	0.32	[− 0.01, 0.64]	0.52	[0.39, 0.66]	0.46	[0.35, 0.58]	0.65	[0.54, 0.77]	**0.65**	**[0.51, 0.80]**^b^
T5	0.26	[0.10, 0.42]	0.65	[0.31, 0.98]	0.58	[0.44, 0.72]	0.45	[0.34, 0.57]	**0.66**	**[0.52, 0.80]**	**0.60**	**[0.45, 0.75]**
T6	0.24	[0.10, 0.38]	0.38	[0.05, 0.71]	0.58	[0.44, 0.72]	0.55	[0.42, 0.67]	**0.69**	**[0.46, 0.92]**	**0.66**	**[0.51, 0.82]**^b^
T7	0.35	[0.19, 0.51]	0.55	[0.21, 0.89]	0.53	[0.39, 0.67]	0.48	[0.36, 0.60]	**0.75**	**[0.52, 0.98]**	**0.61**	**[0.47, 0.76]**
T8	0.24	[0.09, 0.40]	0.52	[0.14, 0.91]	0.53	[0.38, 0.68]	0.47	[0.35, 0.58]	**0.81**	**[0.66, 0.96]**	0.55	[0.40, 0.69]
Average	0.26	[0.14, 0.39]	0.47	[0.17, 0.77]	0.54	[0.42, 0.66]	0.48	[0.38, 0.58]	0.67	[0.56, 0.77]	0.61	[0.48, 0.74]

ES = Effect size (based on latent means, *d*_*av*_). Measurement points affected by school closure or alternating lessons are depicted in bold

^a^The effect size estimates for the first pandemic cohort were obtained from a separate model in which the second pandemic cohort was left out and all latent variable covariances were constrained to be equal across cohorts. Without this approach too many technical issues were observed that prevented normal termination of the estimation process. For more details consult SM 4 in the OSF

^b^Performance gaps are significantly larger than performance gaps in the combined pre-pandemic cohorts (range of *p* values of significant tests was from 0.005 to 0.047)

## Discussion

Our study provides empirical answers to the question of how the pandemic has affected young children in their development of one of the most important academic skills for success in life: reading. We examined the effects of the COVID-19-related school closures on students’ average reading performance, their interindividual differences in reading performance, and on known performance gaps in reading between boys and girls and between immigrant and non-immigrant students in second grade. We investigated these effects by comparing data from the first and second COVID-affected cohorts in the school years 2019/20 and 2020/21 with data from four cohorts prior to the pandemic. For each cohort, we used the same longitudinal assessments with eight points of measurement in each school year to provide a detailed analysis of when and how student achievement differed from what was observed before distant learning was in place. Thus, our study complements previous findings on the impact of COVID-19-related school closures on student achievement by studying the effects on young students at the beginning of their school careers and providing insights into the dynamics during the school year using well-powered longitudinal assessments.

### Interpretation of the main findings

At first glance, our results might be considered promising, as the reading skills of second graders in the first pandemic cohort surprisingly exceeded the average performance of students at the end of second grade before the pandemic, just during and after the first phase of school closures. And, even in the second school year, in which students had experienced up to 14 months of constantly changing learning conditions, the data show that students’ performance was not systematically lower but rather very similar compared to before the pandemic. These findings contradict the common perception that students suffered alarming learning losses due to the pandemic and due to teachers’ inadequate implementation of distance learning. They also contradict the assumption and finding that particularly young students’ academic learning was impaired by the school closures (e.g., Georgiou, 2021; Hammerstein et al., 2021; Tomasik et al., 2021). However, they fit well with some previous studies on the impact of the COVID-19 pandemic on student learning in reading, which also found no systematic adverse effects (Depping et al., 2021; Gore et al., 2021). Overall, the result pattern indicates that benefits for the pandemic cohorts occurred primarily during the periods when the schools were closed, e.g., at the last three points of measurement in the first pandemic cohort and around Christmas in the second pandemic cohort. One possible explanation for this pattern is that parental or other family members support for young students primarily involved reading activities resulting in more reading time at home during these periods (Gore et al., 2021).

A second look at our data, however, reveals a different picture. Schools had not overcome the pandemic without detriments, particularly when it comes to the widening of achievement gaps. While the first pandemic cohort again did not show increased interindividual differences in reading performance, the second pandemic cohort did. Interindividual differences were always larger in the second pandemic cohort, and, at four points of measurement, this increase in variance was significant. This contrasts with the findings of König and Frey's (2022) meta-analysis, which suggests that the negative effects weaken over time. Instead, the fact that the interindividual differences widened especially in the second pandemic year contradict, in our opinion, the assumption that the pandemic had only a short-term negative effect.

Regarding performance gaps between girls and boys, we found that girls significantly outperformed boys at all measurement points, which is in line with findings from previous studies on reading achievement (Mullis et al., 2017). Effect sizes were small (ES ≈ 0.10). However, although girls show advantages in several reading-related variables (e.g., reading motivation, reading behavior) and spend more time reading in their leisure time (Becker & McElvany, 2018; Lepper et al., 2021; McElvany et al., 2017) and during school closures (Grewenig et al., 2020; Wößmann et al., 2020), the achievement gap between boys and girls remained constant across both pandemic cohorts. This is in line with the study by Ludewig et al. (2022), who also found no increase in the gender gap between 2016 and 2021 for fourth graders using the *PIRLS* data.

A particular pattern of results was found for migration background. As expected, achievement gaps were found between immigrant and non-immigrant students with moderate to large effect sizes, and during the first phase of the pandemic, this achievement gap widened and was still large in the second year of the pandemic. This contrasts with the findings of Schult and Wagner (2021), who found that learning loss was not strongly associated with migration background and the results from Ludewig et al. (2022), who found that achievement gaps between students with and without a migration background had not widened in the *PIRLS* data. Ludewig et al. (2022) argued that statistical power was low and that increasing differences for children with and without a migration background would likely be found in longitudinal studies with larger samples. On the one hand, our findings prove them right. On the other hand, however, our detailed data of six different school years also show that these adverse effects for immigrant children were not sudden and were not only detected in the first phase of the pandemic. Instead, the detailed analyses of the achievement gap between immigrant and non-immigrant students in the different pre-pandemic cohorts in our study revealed a general tendency for these gaps to widen in recent years. And although the increase from the school year before the pandemic (2018/19) and the first year of the pandemic was quite large (0.48 vs. 0.66), this increase is by no means unique, as a similarly large increase was observed between the school years 2015/16 and 2016/17 (0.26 vs. 0.47), when a particularly large number of children from foreign countries were admitted to German schools. The finding of an overall increase in achievement gaps between students from different family backgrounds aligns well with PIRLS data, which show that the particularly strong association between student reading achievement and social status has increased in Germany since 2001 (Bos et al., 2017). In our view, the presented data indicate that (a) it is unlikely that the pandemic had no effect on the trend in achievement gaps, as there was a clear increase, especially for the first three to four measurement time points after the school closures compared to the first measurements in the school year 2019/20 and (b) the achievement gap in Germany is widening further and not only the pandemic, but also particular pressures on the school system in general (e.g., admission of many children who do not speak German) are particular drivers of social inequalities. It is not unlikely that the current stress situation of German primary school teachers and the current teacher shortage will exacerbate these problems in the future.

While contradicting previous studies from Germany, our result of increasing interindividual differences and a widening of the achievement gap between immigrant and non-immigrant students is well in line with prior studies showing that effects differ for students depending on their SES (e.g., Donnelly & Patrinos, 2021; Hammerstein et al., 2021). Unfortunately, no data on students’ socio-economic status are available in the quop system. However, it is to be expected that the disadvantageous learning developments will not only affect students with a migration background but will also become apparent for students with low socio-economic status.

Educational attainment determines our opportunities for becoming active and empowered members of the economy and society. It is, therefore, critical to understand how the COVID-19 pandemic has affected education and whether the pandemic has served as a driver of social inequality by increasing educational inequality. Our findings highlight the importance of comparing long-term data from the pandemic to comprehensive pre-pandemic data to differentiate effects of the pandemic from general trends. Interpreting the pattern of results, we conclude that the pandemic in Germany led to an exacerbation of precisely those problems in education that we are already struggling with. As mentioned in the Introduction, the main problem in Germany has not been that students’ overall reading performance is strikingly low, and this is still not the case even after 2 years of a global pandemic. The problem in Germany for more than 20 years has been that interindividual differences in student performance are much larger than in many other European countries and that students’ academic skills are highly related to their socio-economic and migration background. Our data suggest that these very problems have been—at least in part—exacerbated by the pandemic. In contrast, the achievement gap between girls and boys, which is found in almost all countries, and which is not exceptionally large in Germany, was not affected.

### Educational implications

Our results show that in the future, even more than before the pandemic, it will be necessary to deal with the great heterogeneity among students by adapting instruction to students with different learning needs. It is likely that more than before, teachers will experience the whole range of reading competencies in their class: While some children barely know letters and have difficulty synthesizing phonemes, others will read fluently and can comprehend challenging texts without difficulty. Therefore, instruction should address students reading accuracy, reading fluency, and reading comprehension depending on students’ skill level (cf. Förster et al., 2018; Müller et al., 2017). A rational basis to differentiate and adjust instruction is assessment data on student performance, students’ individual development, and thus their response to instruction (e.g., Förster et al., 2018; Hebbecker et al., 2022). This places high demands on teachers. They need professional knowledge about how to conduct such assessments, how to analyze and interpret assessment data, and how to translate assessment information into differentiated instruction for whole classes (e.g., Gelderblom et al., 2016; Zeuch et al., 2017). Moreover, our findings show that special attention should be paid to children with immigrant backgrounds. Schools will need coherent, long-term strategies to adequately nurture the potential of all children.

It is possible, however, that the lessons learned during the pandemic may also provide an opportunity to address these challenges. As it became apparent that the use of digital tools was a prerequisite for distance learning, both teachers and students became more familiar with digital learning environments. This improved the conditions for implementing approaches such as formative assessment or data-driven decision making, which largely require the proficient use of appropriate digital tools to support differentiated instruction in the classroom (e.g., Baron et al., 2019; Souvignier et al., 2021). In addition, implementing innovative instructional approaches to support at-risk students requires a commitment to professional development for teachers. Digital solutions for in-service teacher professional development in the form of video conferences and instructional videos have also increased the chances that teachers will be able to participate in professional development courses.

### Limitations

When interpreting our findings, one needs to consider the learning progress assessments on which they are based. Usually, these assessments are applied in school, but during the school closures, we explicitly asked teachers to instruct their students to take the assessments at home to monitor their learning progress remotely. It is likely that tests were administered at home particularly by students who had better equipment (e.g., a computer, good internet) and more parental support, leading to systematic selection of assessment data. In this context, we would like to point out that the increase in interindividual differences cannot be explained by larger differences in test administration at home. Although test conditions probably differed more for children at home than at school, the data for the last three measurement time points in 2019/20—i.e., during the first lockdown—show a decrease in interindividual differences. It is not until the second school year affected by the pandemic that the increase in interindividual differences becomes apparent. And here the trend is evident at all measurement time points, i.e., even when the tests took place in the schools. In general, we recommend interpreting the overall pattern rather than the individual measurement time points.

To further check the robustness of our findings, we also reran our analyses for the first pandemic cohort using propensity score matching as a different way to cope with the particular large proportion of missing data at the later points of measurement in this cohort (see SM 4). The fact that results were not affected by the way we handled missing data and the fact that measurements were strictly invariant across cohorts strengthen the interpretation of our findings.

All data came from classes in which the learning progress assessment was used even before the pandemic. Therefore, the extent to which the results can be generalized to other classes remains an open question. In some studies, positive effects of school closures on student achievement have been found, and in three of these studies, some kind of learning software was used to assess student achievement (Meeter, 2021; Spitzer & Musslick, 2021; van der Velde et al., 2021). However, the assessments in the present study consist of 46 items, take an average of less than 10 min, and are administered every three weeks. It is thus unlikely that students learned substantially from the assessments.

Clearly, having information about students’ socio-economic background would be useful to better understand our findings. Unfortunately, such data are not available in the quop system. In addition to the students’ gender and date of birth, teachers simply enter whether students have a migration background or special educational needs. The lack of a clear definition of migration background could be regarded as a limitation of our study. In our data, however, teachers recorded a migration background for about 24% of the students. This fits very well with the PIRLS data from 2016, which show that 9.9% of the children had one parent and 15.1% had both parents born abroad (Wendt & Schwippert, 2017). We therefore assume that teachers’ coding of migration background is reliable.

## Conclusion

The results of our study underline the importance of considering potential effects of the pandemic across different cohorts and longitudinally over the course of the school year. While we observed the expected increase in interindividual differences in reading performance and a widening performance gap between immigrant and non-immigrant students, we also found that performance gaps between immigrant and non-immigrant students have widened more or less continuously in recent years. Also, the pandemic did not seem to reduce the overall level of reading performance. The first pandemic cohort was even associated with a higher performance in times of distance learning than in the pre-pandemic cohorts. Future research should further explore context factors that moderate cohort-specific reactions to the pandemic as well as processes such as reading-related leisure activities that might counteract negative consequences of distance learning.

## Footnotes

1 We checked the robustness of our findings by also performing all analyses separately for the word, sentence, and text levels. The results do not differ for the three subscales and are similar to those for the integrated score reported below. Please see the SM 6 for the results at the word, sentence, and text level.
